# Expanding the chemical space of ionic liquids using conditional variational autoencoders

**DOI:** 10.1039/d5sc08673f

**Published:** 2026-01-12

**Authors:** Gaopeng Ren, Austin M. Mroz, Frederik Philippi, Tom Welton, Kim E. Jelfs

**Affiliations:** a Department of Chemistry, Imperial College London White City Campus London W12 0BZ UK k.jelfs@imperial.ac.uk; b I-X Centre for AI in Science, Imperial College London White City Campus London W12 0BZ UK

## Abstract

Ionic liquids (ILs) are salts set apart by their low melting points and can act as highly tuneable solvents with broad application potential, for example as catalysts, in batteries, and for drug delivery. The potential chemical space of ILs is vast, with only a very small region having been explored to date. Machine learning offers a promising approach to advance into this vast space of unexplored ILs; however, existing IL databases contain limited ion diversity, constraining the performance of generative models. To address this, we introduce conditional variational autoencoders (CVAEs) and a novel ion scoring method as a conditioning factor. The ion score prioritises ions with a higher likelihood of forming low-melting-point ILs. Our CVAEs effectively generate novel and diverse cations and anions. Furthermore, we constructed a melting point prediction model to identify cation–anion pairs that are likely to yield ILs with low melting points. Visualisation of the generated ILs alongside existing ones reveals that our approach effectively expands the chemical space of ILs with novel structures. Molecular dynamics simulations further validate that 13/15 of the generated ILs possess desirable low melting points (<373 K). The associated code is available at https://github.com/fate1997/ILGen-ion.

## Abstract 2

Ion-score-guided ion generation, coupled with structure filters and melting point prediction, to expand the chemical space of low-melting-point ionic liquids.

## Graphical abstract

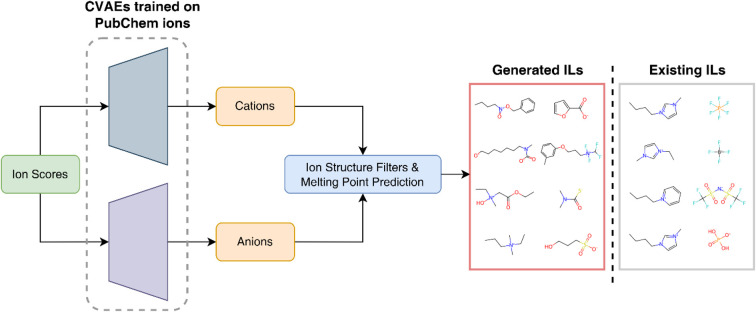

## 1 Introduction

Ionic liquids (ILs) are compounds composed of cations and anions. The main difference between ILs and conventional salts (cation–anion pairs, *e.g.* sodium chloride) is their low melting points, typically below 373 K. Due to the complex interactions between the ions, ILs exhibit a range of unique physicochemical properties, making them valuable in diverse fields,^1^ including separation processes,^2–4^ chemical reactions,^5–7^ and energy systems.^8–10^ Given their nature as cation–anion combinations, ILs possess an expansive chemical space of different cations and anions,^11^ enabling high tunability for specific applications.

Despite the immense potential chemical space of ILs,^12^ the existing chemical space of experimentally validated ILs remains remarkably small, on the order of thousands.^13^ This significant disparity highlights a vast unexplored territory of ILs. Given the impossibility of experimentally validating such a large number of compounds, computer-aided molecular discovery offers an alternative method for expanding the existing IL chemical space.^14^ Traditional approaches, including density functional theory (DFT) and molecular dynamics (MD), provide valuable insights into the atomistic behaviour of ILs and their structure–property relationships. However, these methods are computationally expensive, limiting their scalability for high-throughput screening.^15,16^ Thermodynamic models such as PC-SAFT and COSMO-RS^17–20^ have been widely used for IL property prediction, yet their generalisation to novel and exotic structures remains limited.^21^

In recent years, machine learning (ML) has emerged as a promising avenue for virtual screening. ML has been extensively applied to predict IL properties, including melting point,^22^ viscosity,^23^ and CO_2_ solubility.^24^ ML models offer rapid and accurate predictions, making them highly suitable for virtual screening. In the virtual screening process, it is important to construct a large initial database. One straightforward approach to generating new ion structures involves manually defining a fragment library and then combinatorially combining these fragments. Ion structures can be divided into two parts: the charged components, *e.g.*, imidazolium for cations and carboxylate for anions, and the substituents used to functionalise the charged component, *e.g.*, methyl and halogens. While this method can generate a substantial number of ion structures from pre-defined building groups,^2,10,25,26^ the resulting structures are often highly similar to the original systems, leading to limited expansion of the diversity of IL chemical space. Moreover, many existing melting point prediction models are trained exclusively on IL datasets, which are heavily biased toward low melting points, thereby limiting their effectiveness in screening applications.

Deep generative models are an alternative method to enlarge and diversify molecular chemical space, and they have been widely used in drug discovery^27^ and materials design.^28^ These models generate new samples based on training data. Thus, their performance heavily depends on the quality and quantity of the data in the available database. However, IL databases often suffer from data scarcity, impacting both unlabelled and labelled datasets. This restricts the chemical space of generated ILs and complicates the development of property-guided generative models. To alleviate the data scarcity problem, Liu *et al.*^24,29^ proposed optimisation-based methods to guide generated examples towards higher validity and desired properties. However, due to the limited number of unique cation and anion structures, their generated examples highly resemble the existing ones. Transfer learning provides another strategy to mitigate the data scarcity problem by leveraging knowledge from different but related domains. This typically involves a two-step process: training a large model on a broad database, then fine-tuning it on a smaller, related database. Beckner *et al.*^30^ applied transfer learning to expand the IL chemical space by pre-training variational autoencoders (VAEs) on the GDB-17 database (general organic compounds) and then fine-tuning them on an IL database. They demonstrated that transfer learning is an effective approach to creating a generative neural network model of scarce datasets. However, they also found that the majority of the generated examples are neutral due to the large number of neutral compounds in the pre-training database (GDB-17). More recently, Chen *et al.*^31^ compiled a large, ion database from PubChem^32^ and proposed a pre-trained model for IL property prediction. They further pre-trained a VAE on this database and then fine-tuned it on a labelled IL database.^33^ Their results show that transfer learning can effectively alleviate the data scarcity problem in IL databases. However, this work does not take the melting point into consideration, and so the generated examples are not guaranteed to be low-melting-point ILs. In our previous work,^34^ we applied a link prediction algorithm to address the data scarcity problem and considered melting points explicitly. However, this workflow did not incorporate more ion structures other than the existing IL ions, which limited its ability to generate structurally diverse and novel ions.

Here, we aim to expand the chemical space of ILs with a specific focus on low-melting-point ILs. This requires designing ions with structures dissimilar to those of existing ILs and identifying low-melting-point ILs using a general melting point prediction model. Such an expansion is important not only for computational discovery but also for experimentalists, as it increases the likelihood of identifying novel ILs with unconventional structures and properties, thereby enabling new structure–property analyses and theoretical insights. To achieve this, we first collected large ion databases from PubChem, yielding approximately 0.9 million cations and 0.4 million anions. These PubChem ions cover the existing chemical space of IL ions; however, the vast disparity in quantity between PubChem ions (millions) and IL-specific ions (thousands) significantly reduces sample efficiency in identifying high-quality ions that readily form low-melting-point ILs. To address this and leverage prior knowledge from existing IL databases, we introduce ion scorers. These aim to softly classify whether an ion is likely derived from general ions or those represented in existing ILs. We then trained conditional VAEs (CVAEs) on the general ions using these predicted ion scores as a conditioning factor. After training, we used the ion score as a condition to generate ions that were likely to form low-melting-point ILs. Subsequently, we trained a melting point prediction model on a general melting point database and applied it to identify low-melting-point cation–anion pairs with less bias on underestimation of melting points. Finally, the chemical space visualisation confirmed the effectiveness of our workflow, demonstrating that the generated ILs are clearly distinct from existing ILs. Moreover, MD simulations validate that 13 out of 15 sampled ILs exhibit low melting points (<373 K).

## 2 Methods

### 2.1 Data collection

Detailed information as to the origins of the datasets used is given further below, but here we summarise the key features of the datasets and provide, in bold text, the short-name that will be used to refer to them:

• **PubChem** ion databases: contains cations and anions extracted from PubChem.

• Collected **IL** database: a compilation of ILs gathered from several sources.

• General melting point database (**general MPT**): a comprehensive dataset of melting points, covering a wide range of cation–anion pairs from low-melting-point ILs to high-melting non-IL systems.

• IL melting point database (**IL MPT**): a melting point database for ILs, collected by Venkatraman *et al.*^35^

These datasets were used to train various ML models aimed at generating novel, diverse, and valid ILs. All collected data, along with the full implementation of the methods described in this paper, are available at https://github.com/fate1997/ILGen-ion.

**Fig. 1:**
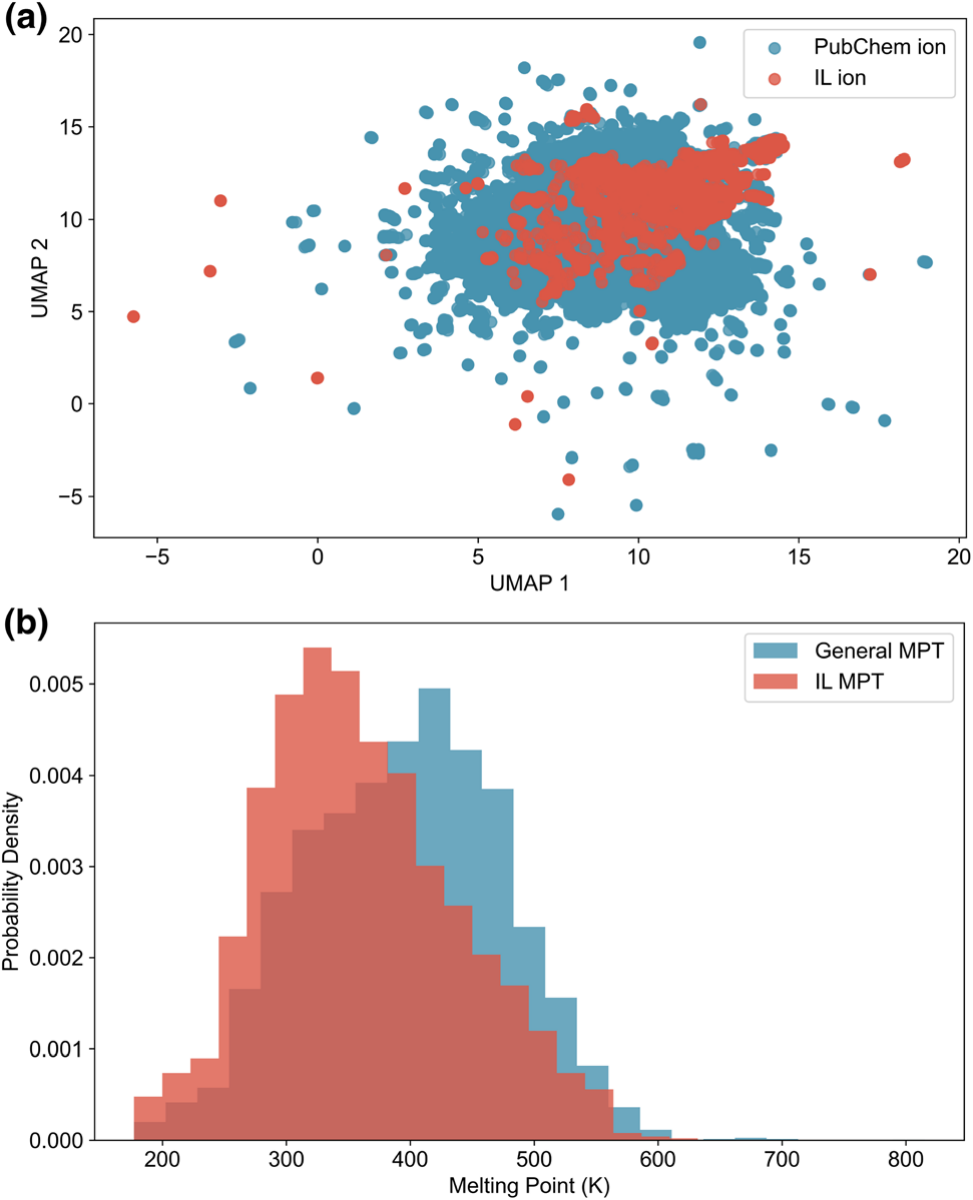
Data visualisation of ions and melting points. (a) UMAP projections of ions from PubChem and ILs. (b) Melting point distributions of the general MPT database (blue) and the IL MPT database (red).

We collected IL ions and PubChem ions to train the conditional generation models. To visualise the chemical space of ions from both PubChem and the IL dataset, we applied the Uniform Manifold Approximation and Projection (UMAP) algorithm^36^ using extended-connectivity fingerprints (ECFPs)^37^ as input features. Owing to the size of the PubChem ion datasets, a random sample of 50 000 cations and 50 000 anions was selected for plotting. As shown in Fig. 1a, the sampled PubChem ions span a broader chemical space than the ions in the IL dataset, demonstrating their potential to enrich the diversity of generated ILs. The specific composition and dataset generation methods and criteria for the IL dataset and the PubChem ion dataset are described in the following sections, Section 2.1.1 and 2.1.2, respectively.

#### 2.1.1 IL dataset

In this work, we constructed a comprehensive IL dataset by collecting and combining IL structures from the literature^38–44^ and the NIST ILThermo database.^13^ Duplicate entries were removed, and molecules were filtered based on the following criteria: (1) failure to be parsed by RDKit, (2) presence of more than two ions, and (3) inclusion of uncommon elements. Elements considered common were H, B, C, N, O, F, P, Cl, Br, I, Li, Na, Al, Si, K, and Fe; all others were treated as uncommon and excluded. Focusing on these common elements can help ensure uniformity and molecular synthesisability within the database. The final dataset comprises 7507 unique ILs, including 3223 distinct cations and 509 distinct anions. It encompasses a broad range of IL families, such as imidazolium, ammonium, pyrrolidinium, sulfonium, and others. Common anions in the dataset include tetrafluoroborate ([BF_4_]^−^), chloride (Cl^−^), and bis(trifluoromethylsulfonyl)imide ([NTf_2_]^−^), among others. The structures and distributions of these ion groups can be found in SI Section S1.

#### 2.1.2 PubChem ions

To generate ILs with diverse ions, a large dataset of cations and anions was collected from PubChem.^45^ To ensure a greater likelihood that synthetically feasible ions are generated, we set some initial selection criteria: anions needed to possess a formal charge greater than −7 and cations a formal charge less than +7; further, only ions with a molecular weight below 500 Da were included. This yielded approximately 1.3 million cations and 0.7 million anions. A subsequent filtering step, adapted from the protocol used for the ZINC dataset,^46^ was then applied. Specifically, molecules were removed if they had calculated log *P* values greater than 6 or less than −4, more than 6 hydrogen-bond donors, more than 11 hydrogen-bond acceptors, or more than 15 rotatable bonds, in order to exclude overly complex ions. Additional filters eliminated molecules that (1) could not be parsed by RDKit, (2) contained uncommon elements, (3) consisted of multiple components, or (4) contained unpaired electrons. These criteria were designed to ensure that the resulting ions were chemically reasonable. After filtering, the final dataset consisted of 903 585 cations and 401 474 anions.

#### 2.1.3 General MPT database

The melting points of ILs can be difficult to determine accurately because glass transitions are common and are particularly sensitive to small amounts of impurities.^47,48^ Consequently, the collected IL melting point (MPT) databases have faced data quality issues.^35^ To address this, we collected melting point data from diverse sources to improve the robustness of our database and excluded entries exhibiting large discrepancies among sources. We compiled a dataset of 5848 melting point values for cation–anion pairs, here referred to as the general MPT database. Melting point data were first collected from diverse chemical databases, including the Bradley dataset,^49^ CRC Handbook,^50^ Wikidata,^51^ CAS Common Chemistry,^52^ the NIST WebBook,^53^ and the Cambridge Structural Database (CSD).^54^ All databases except CSD were accessed using the Chemicals Python package.^55^ As these databases primarily provide CAS Registry Numbers, we converted the CAS numbers to Simplified Molecular Input Line Entry System (SMILES) strings and discarded entries with unparseable CAS numbers. Compounds not comprising exactly one cation and one anion were also removed. In addition, an IL melting point dataset (2206 entries) from Venkatraman *et al.*^35^ was incorporated into the general MPT dataset.

To handle duplicate entries with inconsistent melting points, we applied a filtering rule: if the melting point values differed by more than 10 K, all duplicates were excluded; otherwise, the mean value was used. The melting point distributions for the general MPT and IL MPT datasets are shown in Fig. 1b. The IL MPT dataset comprises a greater number of compounds featuring lower melting points relative to the general MPT dataset, indicating, as expected, that ILs typically possess lower melting points than general cation–anion pairs. In this study, we aim to use the melting point prediction model to identify low-melting-point ILs from general cation–anion pairs. Therefore, relying solely on the IL-specific dataset would risk training a model that underestimates melting points. The general MPT dataset, being larger and more diverse (Fig. S3), provides a more suitable foundation for training a robust predictive model.

### 2.2 Ion scorer

**Fig. 2:**
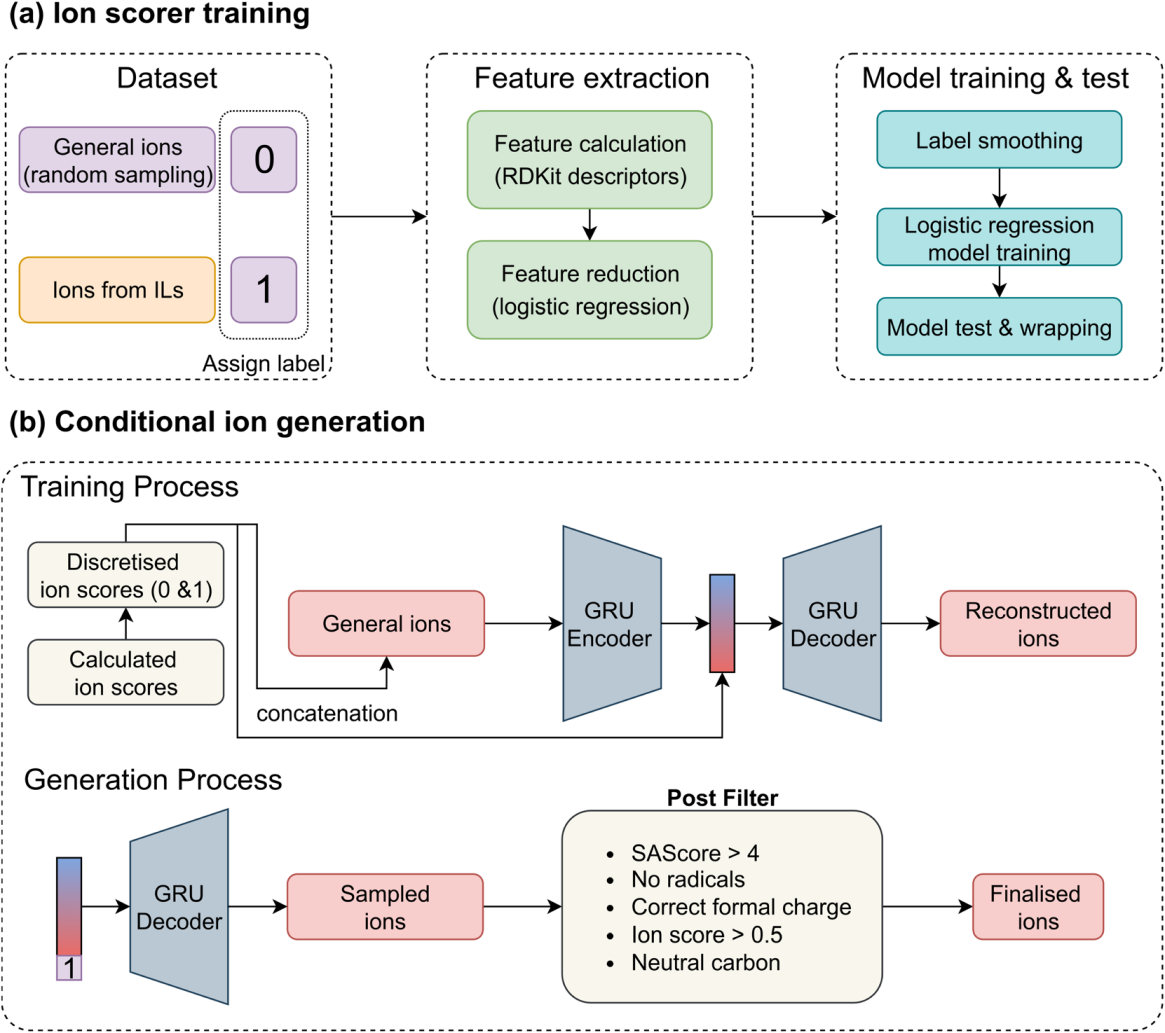
Ion generation. (a) Workflow for training the ion scorers. Ions from the PubChem and the IL databases were labelled as 0 and 1, respectively. RDKit descriptors were computed using the RDKit package and reduced *via* an L1-penalised logistic regression model. Label smoothing was then applied to mitigate overfitting, and a final logistic regression model was trained using the selected descriptors. (b) The conditional generation workflow and model for ion design. Ion scores predicted by the ion scorers were discretised using a threshold of 0.5. These binary labels were concatenated with both the tokenised input sequences and their corresponding latent representations. The trained GRU decoder, conditioned on label 1, was used to generate ions with high predicted ion scores. A post-filtering step was subsequently applied to remove structurally invalid or chemically implausible ions.

A straightforward approach to training an ion generation model would be to collect a broad set of general ions and input them directly into a generative model. However, this strategy overlooks valuable prior knowledge embedded in known ILs – specifically, that certain ions are more likely to form ILs. To fully leverage this information in the conditional generation model, we developed separate ion scorers for cations and anions. These scorers are designed to assign higher scores to compounds resembling ions found in existing ILs, and lower scores to dissimilar ones. These ion scores can help guide generative models toward a more IL-like chemical space. The overall workflow is illustrated in Fig. 2a. We began by assigning binary labels to ions: ions extracted from ILs were labelled as 1, and PubChem ions were labelled as 0. To address the significant imbalance between the number of PubChem (millions) and IL ions (thousands), we randomly sampled an equal number of PubChem ions to match the IL ions, ensuring a balanced dataset that supports building more robust and reliable models. This approach relies on the reasonable assumption that IL ions represent only a small subset of all possible ions, and that a randomly selected PubChem ion is unlikely to be a viable IL ion. For molecular representation, we used RDKit to compute 1D and 2D descriptors, including molecular weight, counts of functional groups, and other chemical features; some of the represented descriptors are shown in SI Section S3. To simplify the model, we performed feature reduction using logistic regression, retaining the 25 most important descriptors. Since the assigned labels may not be perfectly accurate, we employed the label smoothing technique^56^ to prevent the models from over-fitting. Label smoothing modifies the hard labels by introducing a small amount of noise, defined as:

1 *y*_ls_ = (1 − *α*)*y* + *α*/*K*,

where *K* is the number of label classes (2 for binary classification), *α* is a hyperparameter that determines the amount of smoothing, *y* and *y*_ls_ are one-hot labels before and after label smoothing. Here, we set *α* to 0.2 based on ablation studies (SI Section S4). Following label smoothing, the dataset was split into training and test sets in an 80 : 20 ratio. We implemented logistic regression models with L1 regularisation using the scikit-learn Python library.^57^ Finally, the trained ion scorers assign a score (0–1) to ions. We set a threshold of 0.5; ions with scores above 0.5 are classified as IL ions, while those below are classified as non-IL ions (which have a low probability of forming ILs).

### 2.3 Ion generation

After training the ion scorers for both cations and anions, we applied them to all PubChem ions to assign ion scores. Each score was then discretised to 0 or 1 using a threshold of 0.5. Based on these scores, we constructed two CVAEs – one for cation generation and one for anion generation, Fig. 2b. A VAE is a generative model composed of an encoder and a decoder. The encoder maps input data into a latent space, while the decoder reconstructs the data from this latent representation. The latent space is typically regularised to follow a standard normal distribution, allowing for generation from random noise. CVAEs extend this architecture by incorporating auxiliary condition information (here, the ion score) into both the encoder and decoder. The objective of CVAEs is to minimise2
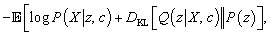
where *X* is the input, *z* is the latent variable, *c* is the condition (ion score), and *P*(*z*) is typically the standard normal distribution. The first term encourages accurate reconstruction, while the second is the Kullback–Leibler (KL) divergence, which regularises the latent space. In our implementation, ions were represented as SMILES strings, linear textual representations of molecular structures. We used Gated Recurrent Units (GRUs) as the encoder and decoder to process these sequences. To incorporate the ion score, we concatenated it with each token embedding in the SMILES during encoding, and also with the latent vector (*z*) during decoding. To improve training stability, we applied a cyclic annealing schedule^58^ to the KL term, gradually adjusting its weight over training epochs. During sampling, a latent vector *z* is drawn from a standard normal distribution, concatenated with a desired ion score (0 or 1), and passed through the trained GRU decoder to generate novel ion structures.

We constructed a vocabulary based on the unique characters in the PubChem dataset. SMILES strings were tokenised into sequences of integers, which were then passed through an embedding layer, producing 292-dimensional vectors. Both the encoder and decoder consisted of three GRU layers with hidden dimensions of 292. The latent space dimensionality was set to 128 (excluding the dimension for the ion score). The models were trained for 100 epochs using the Adam optimiser with a learning rate of 0.0001 and a batch size of 128.

### 2.4 Post-filtering

To ensure the quality of the generated ions, we implemented a post-filtering step based on several criteria. Specifically, we removed ions that: (1) had a synthetic accessibility score (SAScore)^59^ greater than 4, (2) contained unpaired electrons, (3) exhibited incorrect formal charges (*e.g.*, negatively charged cations), (4) received an ion score below 0.5, or (5) contained unstable ion groups, *e.g.*, unstabilised alcoholates and quaternary amides. The representative unstable ion structures are shown in Fig. S6. The unstable ion groups include unstable amides, carbanions, alcoholates and ions with extreme acidity or basicity.

### 2.5 Ion combination & melting point prediction

After generating a large number of cation–anion pairs, it was necessary to identify those most likely to form ILs. A key distinguishing property of ILs is their relatively low melting points. To filter out less promising combinations, we trained a melting point prediction model capable of generalising to both IL and non-IL compounds. For this task, we adopted the TabPFN model,^60^ a transformer-based approach known for its strong performance on tabular data. We configured the model with 16 estimators. For molecular features, we used 197 descriptors computed *via* RDKit.^61^ The general MPT dataset was split into training and test sets using an 80 : 20 ratio. The trained model was subsequently used to screen the generated cation–anion pairs, retaining only those predicted to have low melting points, and thus higher likelihoods of forming ILs.

## 3 Results and discussion

We first present the performance of the melting point prediction models, which are later used to filter cation–anion pairs. Subsequently, we report the performance of the ion scorers and the ion generation model, followed by a detailed analysis of the generated ILs.

### 3.1 Performance of the melting point prediction model

**Fig. 3:**
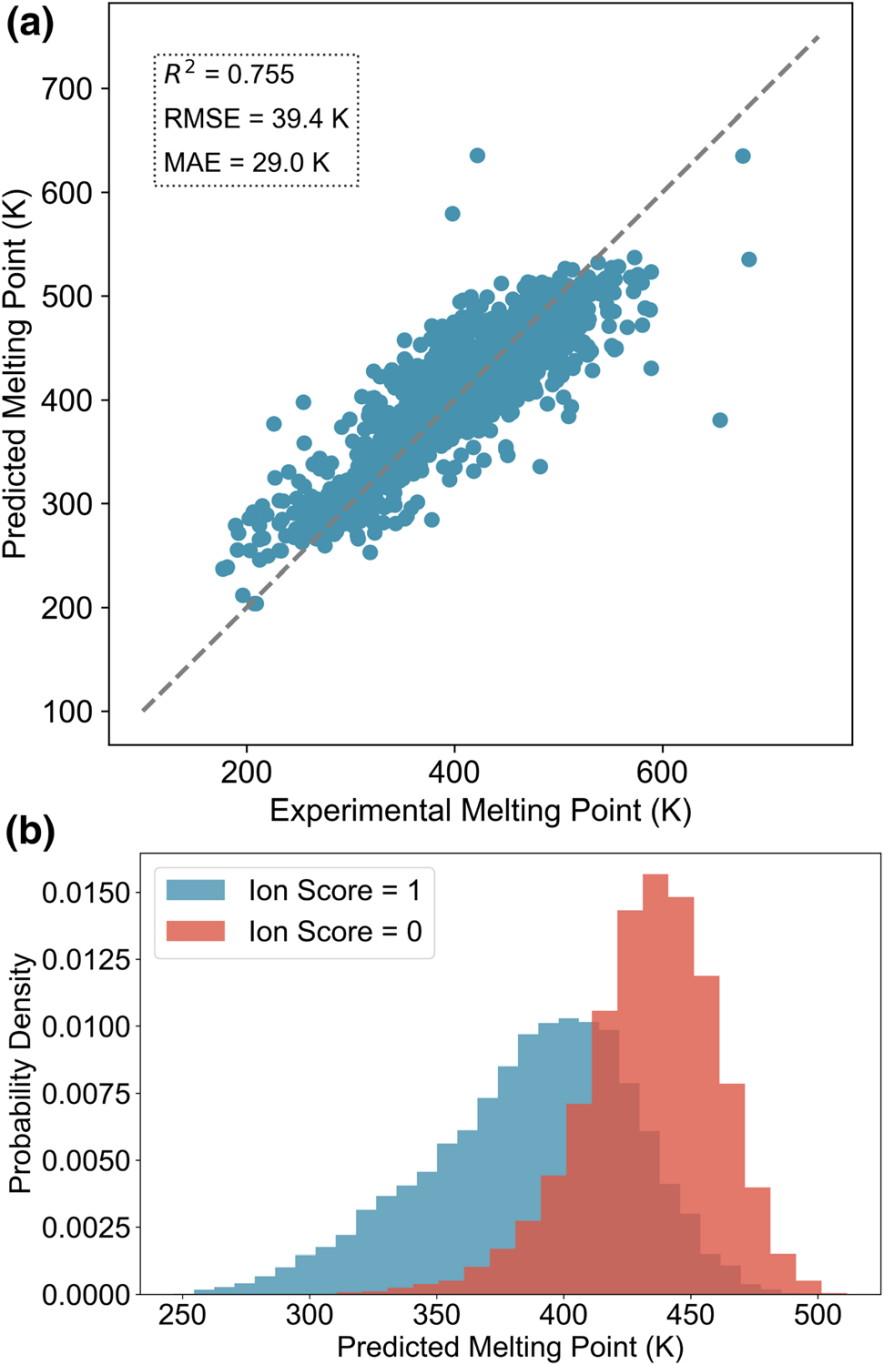
Melting point predictions. (a) Parity plot of predicted *versus* experimental melting points for the test set. (b) Predicted melting point distributions of ILs constructed from ions with an ion score of 1 (high likelihood of forming ILs) *versus* those with an ion score of 0 (low likelihood). Note that these ions are not filtered by the post-filter.

We evaluated the performance of the melting point prediction model using root mean squared error (RMSE), mean absolute error (MAE), and the coefficient of determination (*R*^2^). The parity plot comparing predicted and experimental melting points for the test set of the general MPT database is shown in Fig. 3a. Most data points lie close to the parity line, indicating strong agreement between predictions and experimental values. We also compared the performance of different ML models for melting point prediction, as summarised in Table 1. Models trained on the general MPT database compiled in this work demonstrate competitive performance compared to previously reported IL-specific models. Among the evaluated models on the general database, TabPFN here achieves the best results, with an *R*^2^ of 0.755, an RMSE of 39.4 K, and an MAE of 29.0 K, outperforming both XGBoost and Random Forest (RF). These results highlight the effectiveness of TabPFN in handling complex, tabular molecular data for melting point prediction.

**Table 1:** Performance comparison of different models on melting point prediction^a^

Model	Database size	RMSE (K)↓	MAE (K)↓	*R* ^2^↑
ANN^62^	799	33.3	—b	0.54
RF^35^	2212	45.0	33.0	0.66
KRR^41^	2212	38.5	29.8	0.76
Transformer CNN^63^	3073	45.0	33.7	0.66
GC^64^	3080	37.1	28.8	0.76
XGBoost [this work]	5848	42.5	30.9	0.71
RF [this work]	5848	42.4	31.3	0.72
TabPFN [this work]	5848	39.4	29.0	0.75

a ANN, artificial neural network; RF, random forest; KRR, kernel ridge regression; CNN, convolutional neural networks; XGBoost,^65^ a gradient boosting algorithm for decision trees; and GC, graph convolutional. ↑ indicates “higher is better”, and ↓ indicates “lower is better”.

b The metric is not reported.

Since our dataset includes melting points collected from general MPT databases, it avoids the inherent bias present in IL-specific datasets, which tend to be skewed toward lower melting points (Fig. 1b). ML models trained solely on IL databases are likely to underestimate the melting points of cation–anion pairs, making them less suitable for use as filters to eliminate high-melting-point candidates. In contrast, the general MPT database compiled in this work provides a more balanced and comprehensive view of cation–anion combinations. As a result, models trained on this broader dataset should exhibit reduced bias and be better suited for accurately identifying high-melting-point compounds. This characteristic is particularly valuable in large-scale virtual screening tasks, where the model must generalise well across a diverse chemical space.

### 3.2 Performance of the ion scorers

To maintain simplicity and interpretability in the ion scorers, we performed feature reduction on the RDKit descriptors. Feature importance was evaluated based on the absolute values of the coefficients from a logistic regression model. The 25 most informative descriptors were selected for use in the final models. As illustrated in Fig. S4, the selected descriptors capture critical molecular properties, including structural complexity, hydrogen bonding capability, polarizability, electrostatic interactions, and topological features, all of which are highly relevant to the behaviour of ILs. For instance, hydrogen bonding is known to play a significant role in determining IL properties.^66^ By considering these diverse descriptors, we can gain multiple perspectives and effectively differentiate between PubChem ions and those specifically relevant to ILs.

After feature selection, the logistic regression models were retrained using the top 25 descriptors. The performance of the classification model was measured by accuracy, recall and the area under the receiver operating characteristic curve (ROC-AUC). Accuracy is the proportion of total correct predictions made by the model. Recall is the proportion of actual positives that were correctly identified by the model. ROC-AUC indicates how well the model distinguishes between positive and negative examples, with higher values meaning better performance. Performance metrics for both cation and anion scorers are summarised in Table 2. Despite the model's simplicity, both scorers achieved strong performance, indicating that the classification task is relatively tractable. High recall scores demonstrate the models' effectiveness in identifying IL-relevant ions. Notably, the cation scorer outperformed the anion scorer, likely due to the greater number of cation samples available during training.

**Table 2:** The performance of ion scorers. ↑ indicates “higher is better”

Metric	Cation	Anion
Accuracy↑	0.9147	0.8578
ROC-AUC↑	0.9142	0.8617
Recall↑	0.9511	0.9271

**Fig. 4:**
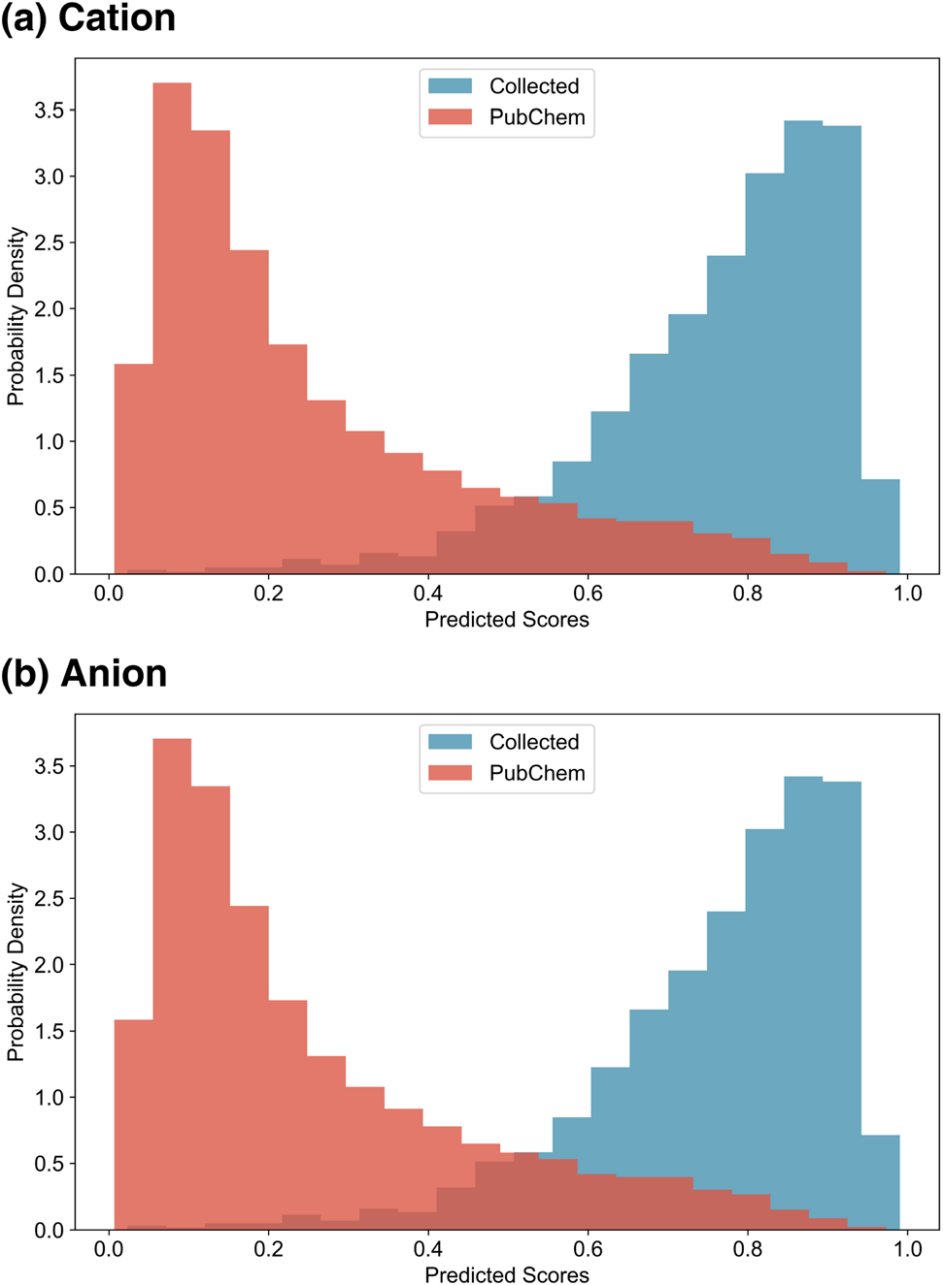
Ion score distributions. (a) Distribution of ion scores for IL cations and PubChem cations. (b) Distribution of ion scores for IL anions and PubChem anions.

After training the ion scorers, we randomly sampled 10 000 PubChem cations and anions and computed the ion scores of PubChem ions and IL ions. The resulting score distributions are presented in Fig. 4. As expected, the scorers successfully assigned higher scores to IL ions and lower scores to PubChem ions, demonstrating effective discrimination between the two classes. Notably, due to the application of label smoothing during training, the score distributions are less sharply polarised. This allows a subset of PubChem ions to receive relatively high scores, reflecting the model's capacity to recognise potentially IL-like structures beyond those present in the training data.

### 3.3 Performance of the ion generation models

We built two separate CVAEs for cations and anions, respectively. To evaluate the performance of the ion generation models, we sampled 10 000 SMILES and assessed them using four metrics: validity, uniqueness, novelty, and reconstruction accuracy. Validity refers to the proportion of generated SMILES that can be parsed by RDKit. Uniqueness measures the fraction of unique SMILES among the valid ones. Novelty quantifies the percentage of generated SMILES not present in the training set. Reconstruction accuracy is defined as the proportion of test set SMILES that are correctly reconstructed by the model. The performance metrics are summarised in Table 3. The cation and anion generation models demonstrate high uniqueness and novelty, indicating their ability to generate diverse and previously unseen structures. However, the validity is not very high, likely due to the complexity of learning syntactic rules from highly diverse ion structures. To further assess the conditional generation capability, we sampled 5000 ions conditioned on label 0 (non-IL ions) and another 5000 on label 1 (IL ions) and calculated their ion scores. The label 1 condition is intended to bias the generation toward ions with a higher likelihood of forming an IL. The average ion scores for label 1 samples were 0.45 for both cations and anions, whereas the averages for label 0 were 0.17 and 0.20, respectively. These results confirm that the conditional generation model effectively produces ions with higher predicted relevance to ILs when guided by label 1. It is worth noting that the generated examples for label 1 do not consistently achieve very high ion scores. This may be due to class imbalance in the training data. For cations, there are 678 076 label 1 examples compared to 176 279 label 0 examples. A similar trend is observed for anions, with 290 998 label 1 examples and 102 235 label 0 examples. This imbalance makes it difficult for the CVAEs to achieve high average scores for label 1. Despite this, the use of ion scores still helps the CVAEs generate more positive examples (0.45 *vs.* 0.20).

**Table 3:** The performance of the CVAEs (recon.: reconstruction accuracy)

Ion type	Validity	Uniqueness	Novelty	Recon.
Cation	77%	100%	99%	71%
Anion	72%	100%	98%	71%

**Fig. 5:**
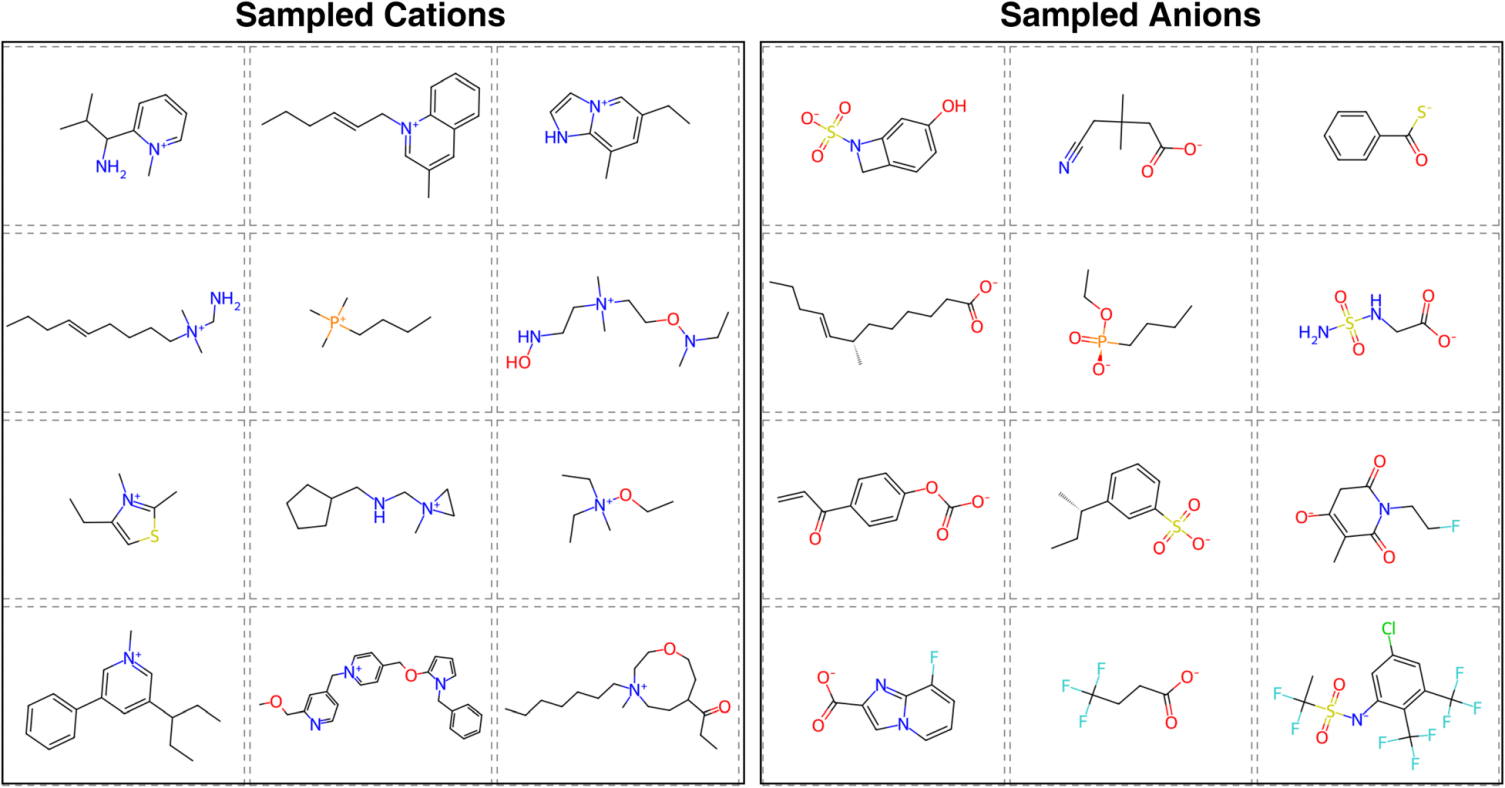
Sampled cations and anions. The cations and anions were generated with label 1 (high likelihood of forming ILs) as the condition. A post filter was applied to the sampled cations and anions to filter out chemically infeasible ions.

Upon visual inspection of the generated ions presented in Fig. S6, we observed that although the ion generation model successfully produces diverse and novel structures, some generated ions exhibit chemically unstable features. For instance, certain ions are excessively complex, contain implausible substructures (*e.g.*, carbanions), or possess radical electrons. There are generally two strategies to address such issues: (1) pre-filtering the training data to exclude undesired structures before model training, or (2) post-filtering the generated molecules to remove invalid or implausible candidates. To better explore potential IL ions across a broad and diverse chemical space, we opted for the post-filtering approach. As described in Section 2.4, we applied several structural and chemical filters to eliminate unreasonable ions from the generated set. Representative examples of filtered ions are shown in Fig. 5. These examples demonstrate that the ion-level generation model produces diverse ion structures. For instance, the positively charged groups include rings of various sizes, ranging from 3-membered to 9-membered rings; while the negatively charged groups include carboxylates, thiocarboxylates, phosphonates, and amides. At the same time, key characteristics commonly found in ILs, such as long alkyl chains and fluorine atoms, are also present in many generated structures. This suggests that the generative model, combined with ion scoring and post-filtering, is capable of exploring a large chemical space while still capturing important structural motifs observed in real ILs.

To further assess the impact of ion scores on the CVAEs, we randomly sampled 100 cations and 100 anions from the decoder, each with ion scores of 0 and 1. Cations and anions with the same ion score were then paired to construct IL candidates. Using the trained melting point prediction model, we estimated the melting points of these ILs, and the results are shown in Fig. 3b. The predicted values indicate that ILs composed of ions with an ion score of 1 generally exhibit lower melting points compared to those formed from ions with a score of 0. This suggests that the ion scorers effectively guide the generation process toward chemical space regions more likely to correspond to low-melting-point ILs. By using ion scores as conditions, the CVAE model is able to generate ions with properties similar to those found in known ILs, increasing the likelihood of forming ILs with desirable melting behaviour. Compared to transfer learning approaches, this conditional generation method offers a softer constraint, and it does not force the generated chemical space to closely mimic the existing IL dataset.^33^ Instead, it allows for meaningful expansion of the IL chemical space while still preserving key characteristics of known IL ions. Overall, the cation and anion CVAEs effectively alleviate the limited diversity of the existing chemical space of ILs.

### 3.4 Performance of generated ILs

**Fig. 6:**
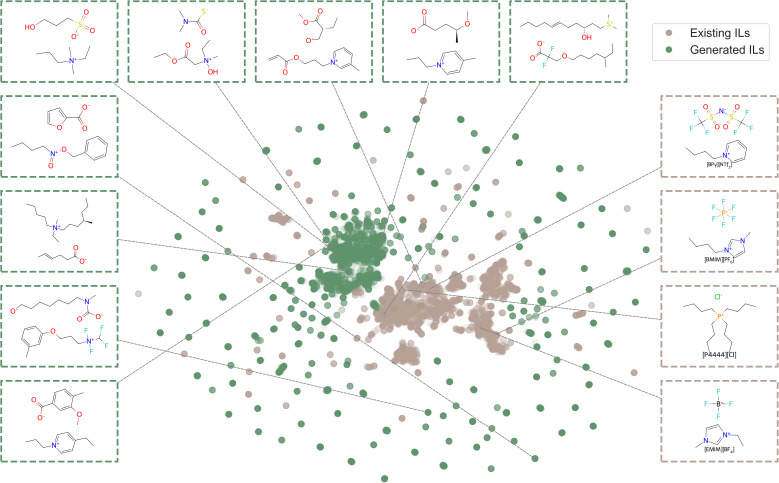
IL chemical space. We calculated ECFP for the generated ILs and existing ILs, and used UMAP to project ILs to a 2D space. We show a few existing IL samples and the generated IL samples from this work. The molecules with green frames are from generated ILs. The molecules with brown frames are from existing ILs.

In addition to the previously sampled ions, we randomly sampled another 100 cations and 100 anions, each conditioned on an ion score of 1. These filtered ions were then combined to generate 10 000 unique ion pairs, which were subsequently evaluated using the melting point prediction model and ranked based on their predicted melting points. The top 5000 ILs with the lowest predicted melting points were selected as the generated IL candidates. As shown in Fig. 6, our workflow effectively expands the existing IL chemical space by discovering numerous novel ILs that are chemically diverse and distinct from those currently known, while also generating ILs similar to existing ones. The divergence in chemical space observed in the generated ILs may be attributed to the fact that the collected ILs database was not directly incorporated into our workflow. Instead, the use of ion scorers as a soft constraint guided the generation process toward a distinct yet chemically plausible region, that is likely to form low-melting-point ILs. Compared to previous IL generation workflows,^24,29^ which primarily focus on ion generation using only known IL ions as inputs, our approach leverages a large pool of PubChem ions to explore a much broader chemical space, enabling the generation of novel ions beyond the scope of existing IL datasets.

Additionally, our framework explicitly incorporates melting point consideration during IL generation, enabling the identification of low-melting-point candidates. We computed the average melting point of the generated ion combinations *via* the trained melting point prediction model, finding it to be approximately 380 K. This is lower than the average melting point of the general MPT database (395 K), indicating that the CVAE models are capable of generating ions that can form lower-melting-point ion mixtures even without melting point filtering. To further validate the effectiveness of our approach, we applied the MD-based workflow from our previous work^34^ to compute the melting points of top 15 generated ILs with the lowest predicted values (details provided in the SI Section S6). The results show that 13 out of 15 ILs exhibit melting points below 373 K, with an average melting point of 353 K. This provides additional confirmation that our workflow can reliably identify low-melting-point ILs.

## 4 Conclusion

We have proposed an ML-based workflow to explore and expand the chemical space of ILs. By leveraging extensive ion databases from PubChem and incorporating ion scorers, we trained CVAEs capable of generating diverse and novel ion structures likely to form low-melting-point ILs. Here, ion scorers aim to assign high scores to ions that are more likely to form ILs. A melting point prediction model, trained on a general melting point dataset, was used to filter out cation–anion pairs with undesirably high melting points. Our workflow fundamentally differs from existing IL design approaches, which either generate IL databases through motif manipulation, producing structures highly similar to known ILs, or rely on limited IL datasets, resulting in biased predictions. The results demonstrate that our framework not only expands the chemical space of existing ILs, but also ensures the generation of ILs with desirable low melting points. This success can be attributed to three key components: (1) the ion scorers, which capture intrinsic features distinguishing IL ions from general ions and assign higher scores to IL-like candidates; (2) the CVAE models, which generate ions conditioned on these scores to favour IL-relevant structures; and (3) the melting point prediction model, trained on a diverse general MPT database, which contains the melting points from a wide range of cation–anion pairs, including low-melting-point ILs and high-melting systems. Some of the representative generated IL examples are shown in SI Section S6.

Looking ahead, promising directions include the direct generation of ion pairs guided by melting-point prediction, the integration of active learning with automated melting-point calculations or experiments, and optimisation-based strategies (*e.g.*, Bayesian optimisation or reinforcement learning) to identify low-melting-point ILs within large chemical spaces more efficiently. We also observed structural imbalances in the PubChem ion dataset; for instance, most anions contain carboxylate functional groups, while others, such as borate-based ions, are underrepresented. This imbalance can limit the diversity of generated ions. Although ion scoring helped mitigate this issue, future work could explore additional strategies, such as data augmentation, to address dataset biases. Meanwhile, we found that several generated ions contained unstable structures. Although we attempted to remove these structures using post-filtering, this approach was not very efficient. We also tested p*K*_a_ prediction models to identify unstable ions; however, this method depends heavily on the accuracy of the prediction model, and the existing models cannot give good p*K*_a_ predictions on IL ions. Overall, our framework, which integrates structural scoring, conditional generation, and predictive filtering, shows promising performance in IL discovery. Furthermore, it holds promise for generalisation to other material systems, such as deep eutectic solvents and transition metal complexes.

## Author contributions

G. R. designed the workflow, conducted experiments, and analysed the results. A. M. M. contributed to the project design and execution. F. P. and T. W. contributed to project conceptualisation. K. E. J. supervised the project. G. R. drafted the manuscript, and all authors contributed to the final version.

## Conflicts of interest

There are no conflicts of interest to declare.

## Supplementary Material

SC-OLF-D5SC08673F-s001

## Data Availability

All data used in this study, including the PubChem, IL, general MPT, and IL MPT databases, along with the code for training the ion scorers, CVAEs, melting point prediction model, and post-filtering procedures, are publicly available at ILGen-ion: https://github.com/fate1997/ILGen-ion. Supplementary information (SI): including IL dataset visualisations, melting point database visualisations, feature importance analyses, ablation studies for ion scorers, chemically unstable ions, and molecular dynamics validation results. See DOI: https://doi.org/10.1039/d5sc08673f.
