# Supplementary material for: Expanding the chemical space of ionic liquids using conditional variational autoencoders
Source: Chem Sci. 2026 Jan 12;17(9):4621–31. doi: 10.1039/d5sc08673f (PMC12810713; doi:10.1039/d5sc08673f)
Supplement: SC-017-D5SC08673F-s001 [file SC-017-D5SC08673F-s001.pdf]

Supplementary Information (SI) for Chemical Science.
This journal is © The Royal Society of Chemistry 2026

Supporting Information: Expanding the chemical space of ionic liquids via conditional variational autoencoders

Gaopeng Ren,[†] Austin Mroz,[†,‡] Frederik D. Philippi,[†] Tom Welton,[†] and Kim E. Jelfs[*,†]

†*Department of Chemistry, Molecular Sciences Research Hub, Imperial College London, White City Campus, Wood Lane, London, W12 0BZ, U.K.*

‡*I-X Centre for AI in Science, Imperial College London, White City Campus, Wood Lane, London, W12 0BZ, U.K.*

E-mail: k.jelfs@imperial.ac.uk

Contents

S1 IL dataset **S-3**

S2 Melting point databases **S-4**

S3 Feature importance **S-5**

S3.1 Feature description S-6

S4 Ablation studies for ion scorers **S-8**

S5 Chemically unstable ions **S-9**

S6 Molecular dynamics validation **S-10**

References **S-15**

S1 IL dataset

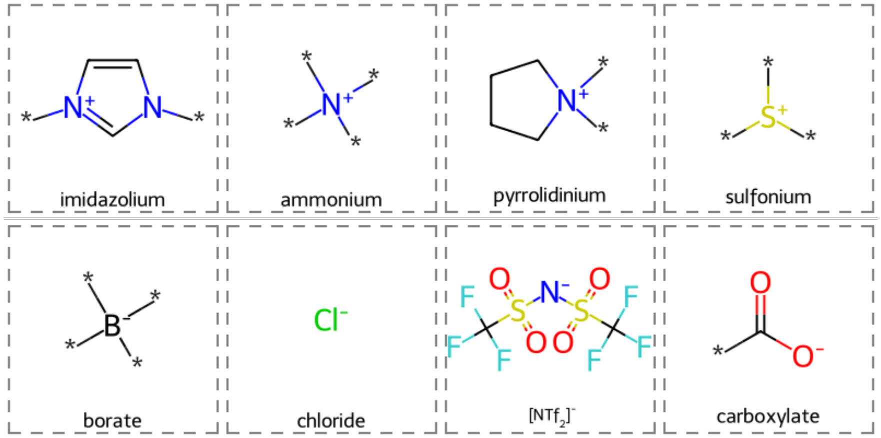

Figure S1: Common cation and anion core groups present in the collected IL dataset. "⋆" indicates potential functionalisation sites.

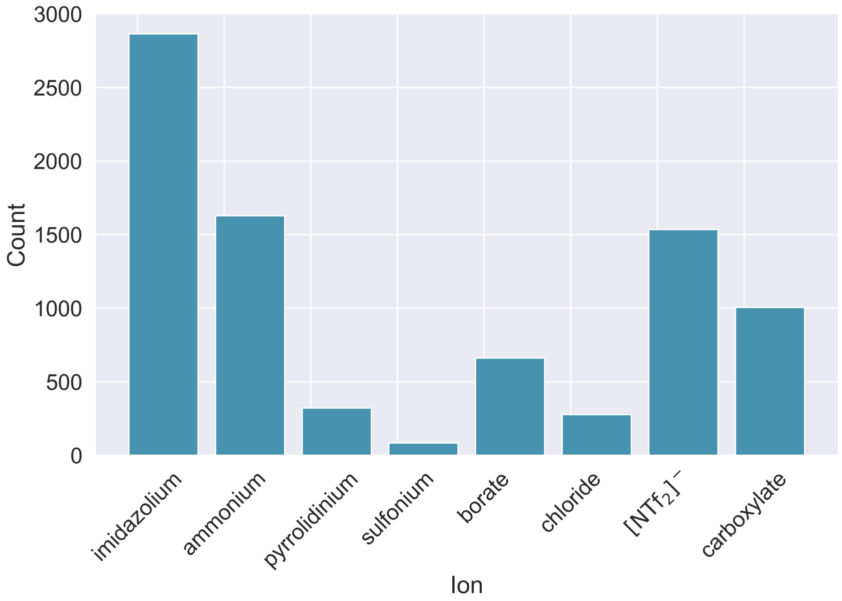

Figure S2: Distribution of common ion core groups in the IL dataset.

S2 Melting point databases

As shown in Figure S3, the general melting point database spans a broader chemical space than the IL melting point database, highlighting its potential to enhance the reliability and generalisation ability of melting point prediction models. Notably, the general melting point database includes data points from the IL melting point database; therefore, some outliers present in the IL database are also captured in the general database.

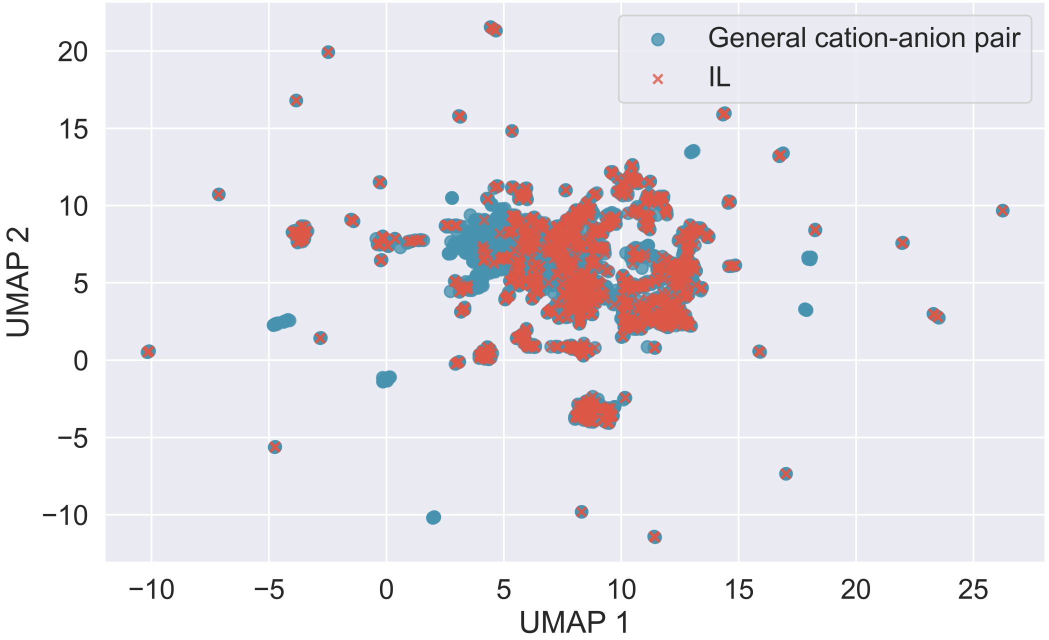

Figure S3: UMAP projections of cation-anion pairs from the general melting point database and the IL melting point database. The UMAP projections are computed using ECFP features as input.

S3 Feature importance

The top 25 most important features for the cation and anion scorers are shown in Figure S4. The feature acronyms are described in Section S3.1.

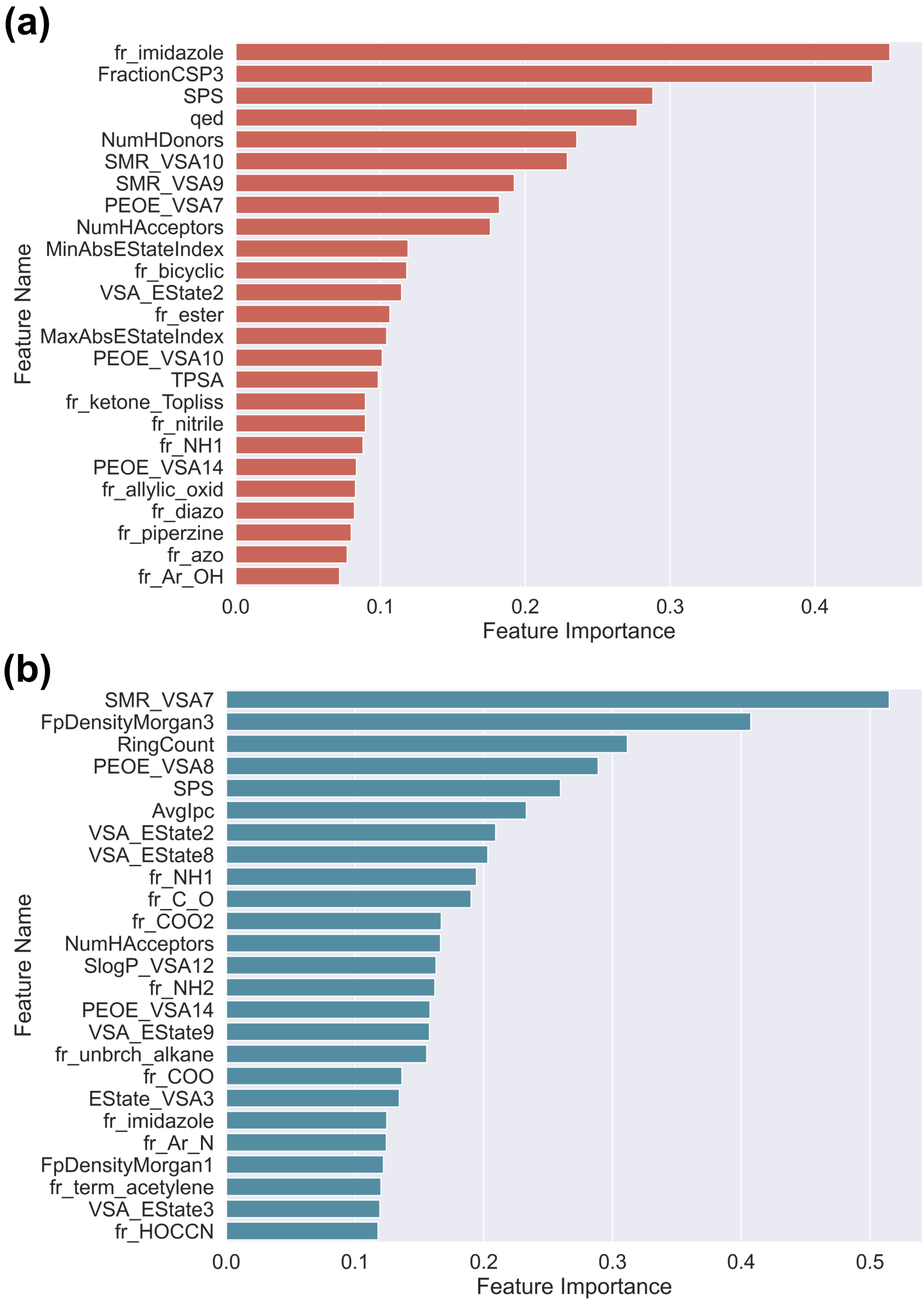

Figure S4: Feature importance ranking for ion scorers. **(a)**. Top 25 important features for the cation scorer, **(b)**. Top 25 important features for the anion scorer.

S3.1 Feature description

The meaning of feature names is:

- FractionCSP3: The fraction of SP3 hybridized carbon atoms.
- qed: Quantitative estimation of drug-likeness.
- SPS: Spacial score.[S1]
- TPSA: Topological polar surface area.[S2]
- NumHDonors: Number of hydrogen donors.
- NumHAcceptors: Number of hydrogen donors.
- SMR_VSA: Polarisability descriptors, related to van der Waals surface area and molar refractivity.[S3]
- Chi2v: Topological descriptors that capture information about the molecule's structure by considering the connectivity of atoms.
- MaxEStateIndex: Maximum electrotopological-state index.
- MinAbsEStateIndex: Minimum absolute electrotopological-state index.
- MaxAbsEStateIndex: Maximum absolute electrotopological-state index.
- FpDensityMorgan3: The density of Morgan fingerprint with radius 3.
- AvgIpc: The average information content of the coefficients of the characteristic polynomial of the adjacency matrix of a hydrogen-suppressed graph of a molecule.
- SlogP_VSA: Descriptors intended to capture hydrophobic and hydrophilic effects either in the receptor or on the way to the receptor.[S3]
- PEOE_VSA: Descriptors intended to capture direct electrostatic interactions.[S3]

- MolWt: Molar weight.
- fr_imidazole: Number of imidazole rings.
- fr_bicyclic: Number of bicyclic rings.
- fr_NH2: Number of primary amines.
- fr_ester: Number of esters.
- fr_ketone_Topliss: Number of ketones excluding diaryl, a,b-unsat. dienones, heteroatom on Calpha.
- fr_nitrile: Number of nitriles.
- fr_NH1: Number of Secondary amines.
- fr_allylic_oxid: Number of allylic oxidation sites excluding steroid dienone.
- fr_diazo: Number of diazo groups.
- fr_piperzine: Number of piperzine rings.
- fr_azo: Number of azo groups.
- fr_Ar_OH: Number of aromatic hydroxyl groups.
- fr_Ar_N: Number of aromatic nitrogens.
- fr_term_acetylene: Number of terminal acetylenes.
- fr_HOCCN: Number of C(OH)CCN-Ctert-alkyl or C(OH)CCN cyclic.
- fr_unbrch_alkane: Number of unbranched alkanes of at least 4 members (excludes halogenated alkanes).
- fr_C_O: Number of carbonyl O.
- fr_COO2: Number of carboxylic acids.

S4 Ablation studies for ion scorers

As shown in Figure S5, when $\alpha = 0.0$ (i.e., no label smoothing is applied), the distributions are highly polarised, with only a small number of PubChem ions classified as IL ions. When $\alpha > 0.0$, the distributions become less polarised. The results show that when $\alpha = 0.2$, the recall is the highest. Therefore, we choose $\alpha = 0.2$ in this work.

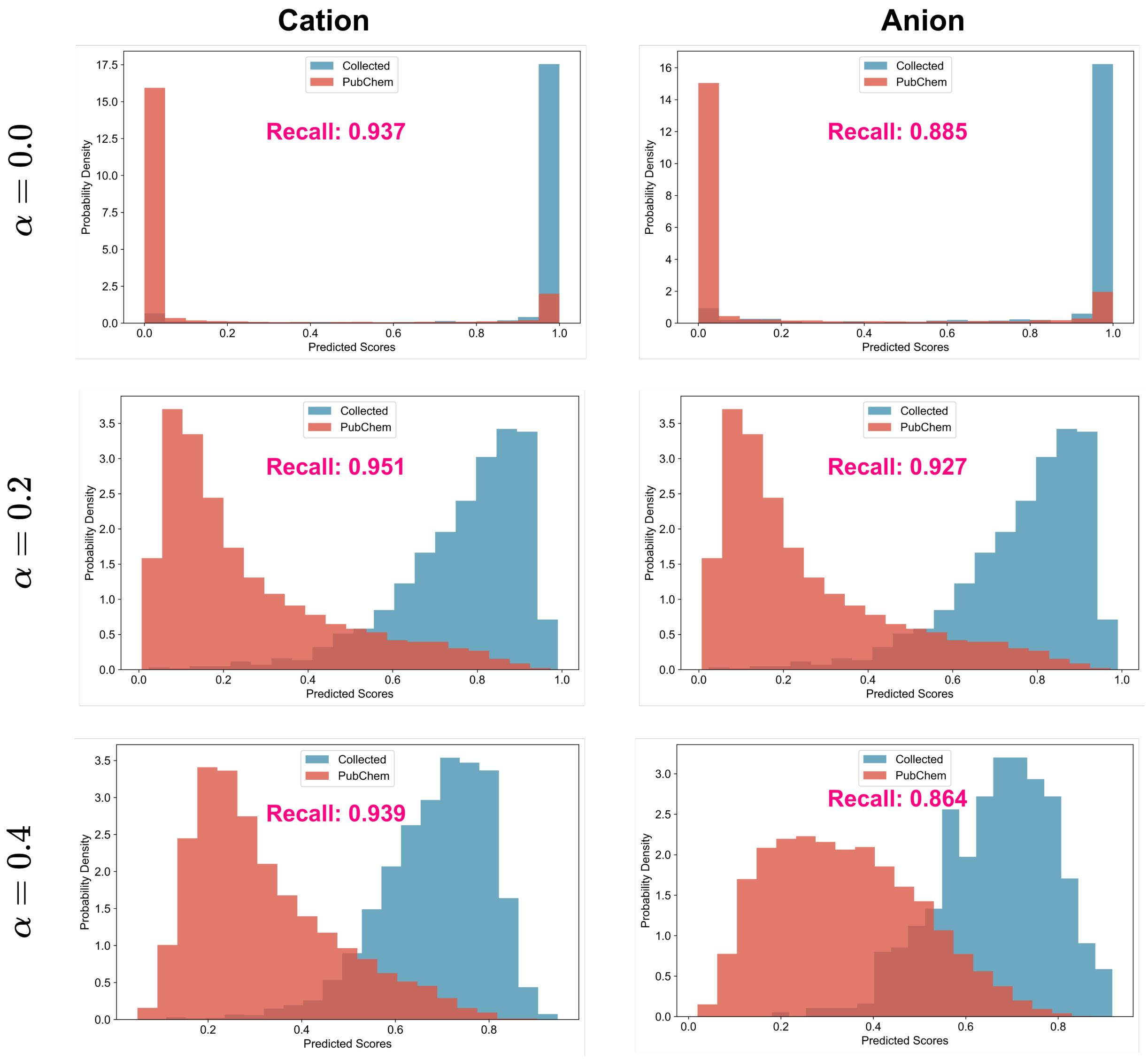

Figure S5: Distribution of ion scores for IL ions and PubChem ions. Each row corresponds to a different α value used in label smoothing, and each column represents either cations or anions. The recall metric is indicated in each subplot.

S5 Chemically unstable ions

Figure S6 shows examples of chemically unstable ions generated by the trained cation and anion CVAEs. These structures contain either radical electrons or unstable ion groups (including unstabilised amide and alcoholate), both of which are associated with high energy and instability. Therefore, a post-filtering step was applied to remove ions featuring such groups/properties from the sampled set.

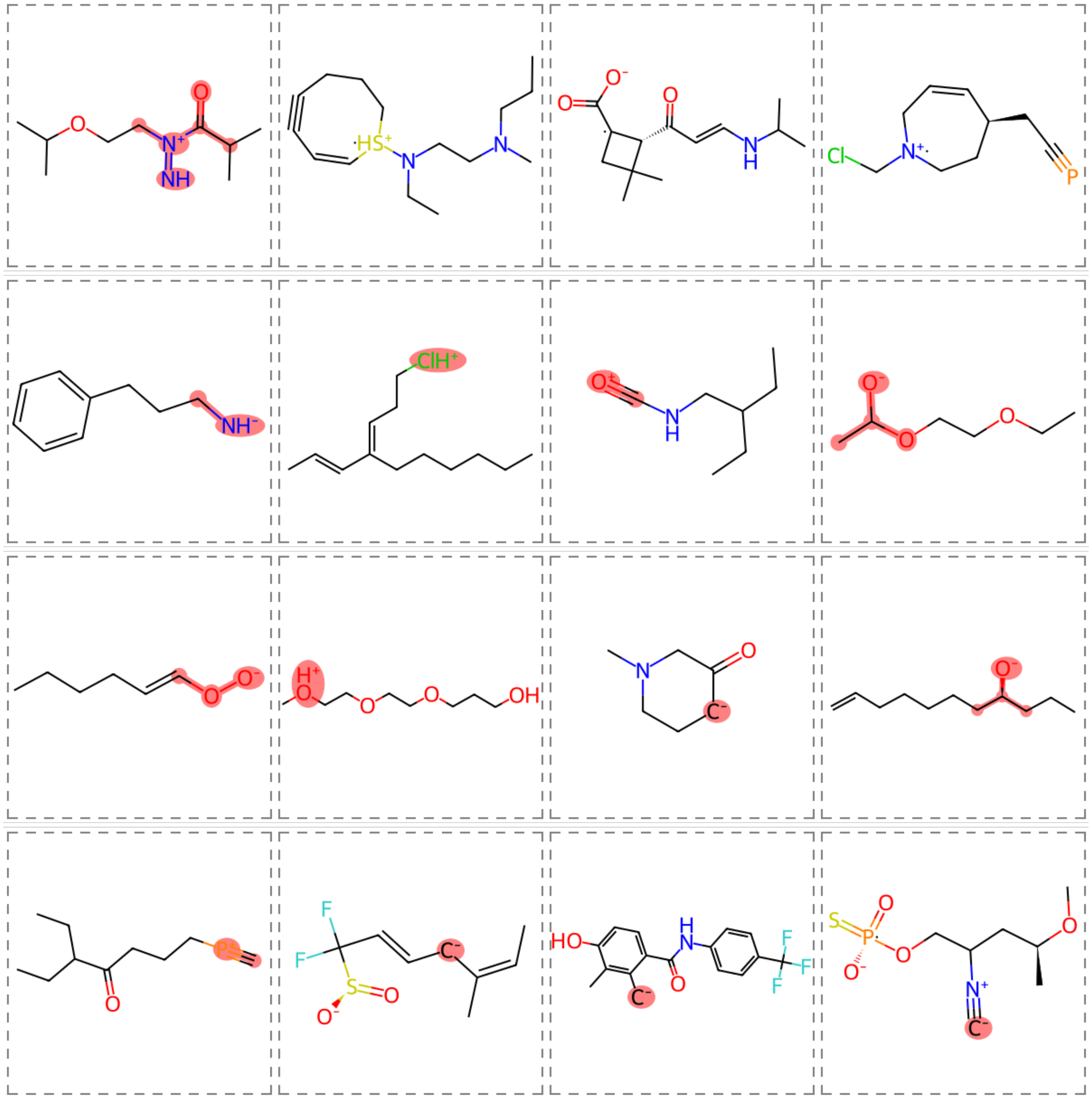

Figure S6: Example chemically unstable cations and anions sampled from the cation CVAE and anion CVAE. The unstable substructures are highlighted with red colour.

S6 Molecular dynamics validation

In this work, we adopt the molecular dynamics workflow from our previous study.[S4] The workflow begins with SMILES strings for a cation and an anion. The corresponding 3D structures are generated using xTB calculations.[S5] Initial configurations of the IL systems were constructed using Packmol,[S6] and the solid phases packed by applying Coulombic potential wells. Annealing simulations are then carried out from 175 K to 600 K using the GROMACS software package. The General AMBER Force Field (GAFF) was employed in the MD simulations. Finally, melting points were estimated based on the curve of approximated diffusion coefficients over simulation time.

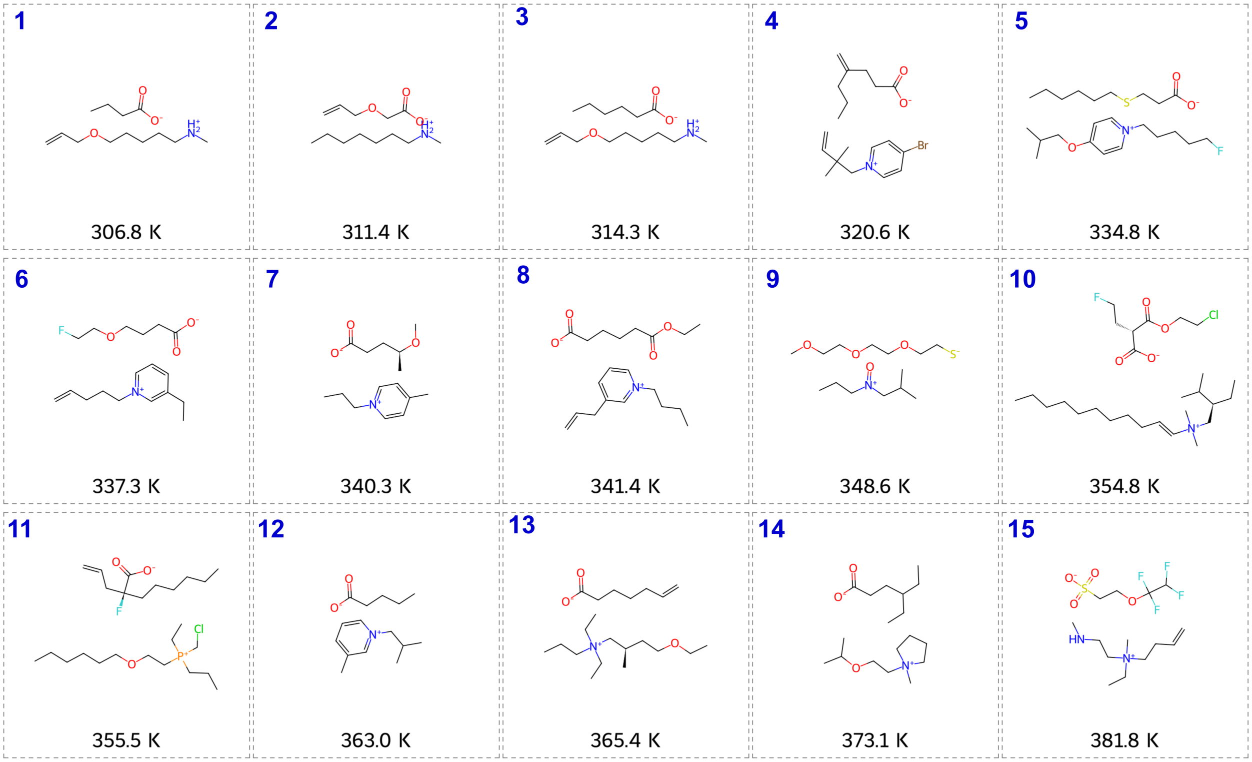

Figure S7: Melting point estimations from molecular dynamics simulations for 15 generated ILs. The ILs are sorted by their MD-estimated melting points.

As shown in Figure S7, the sampled ILs exhibit low estimated melting points (the corresponding plots to determine melting points are shown in Figures S8-S10). Only two ILs have estimated melting points exceeding 373 K, and their melting points remain close to this

threshold (373.1 K and 381.8 K). These results demonstrate the effectiveness of our workflow in generating ILs with low melting points.

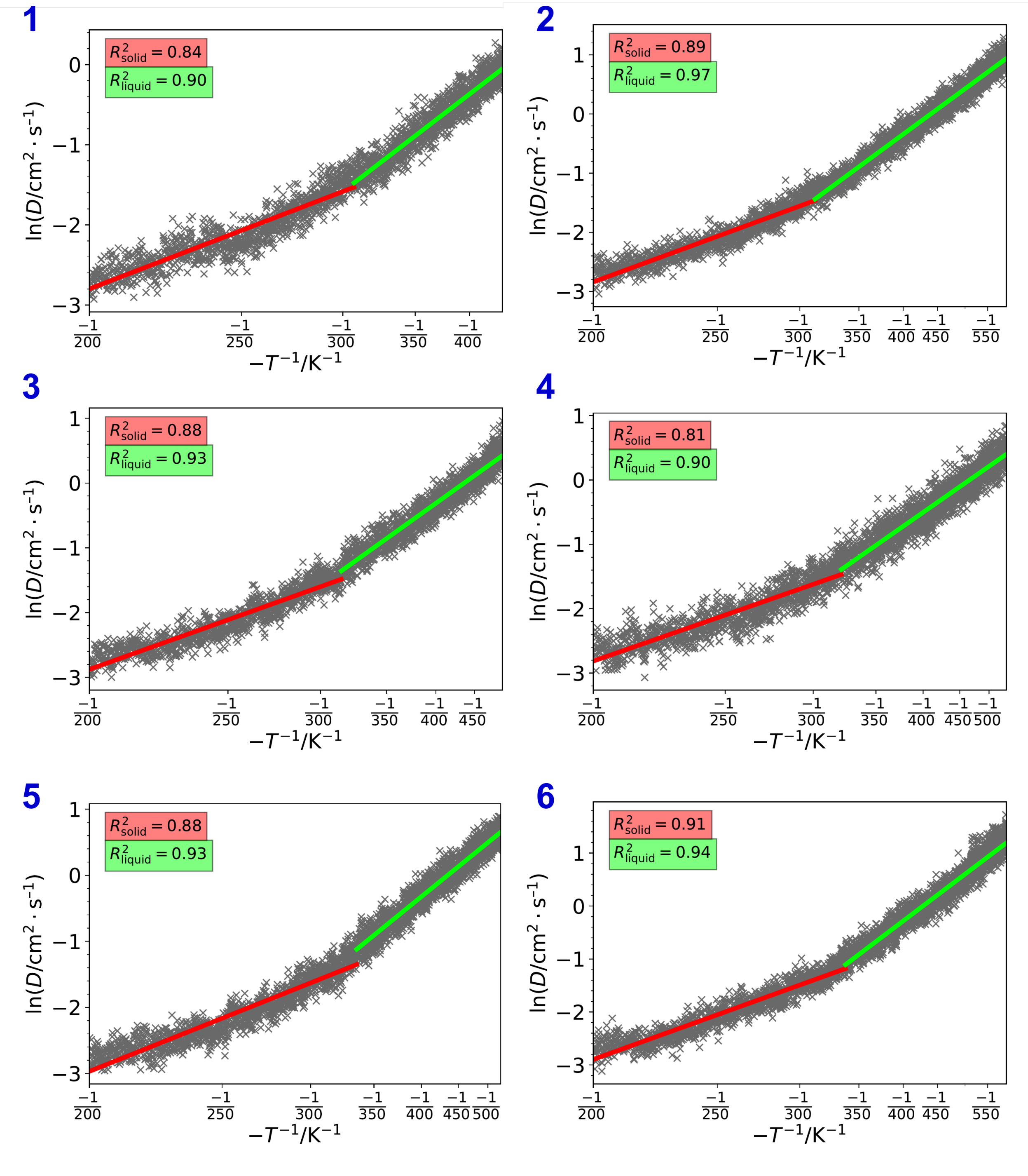

Figure S8: The diffusion coefficient (D) dependence on temperature (T) during annealing simulation for expanded ILs 1-6. The left-top number indicates the corresponding molecules in Figure S7.

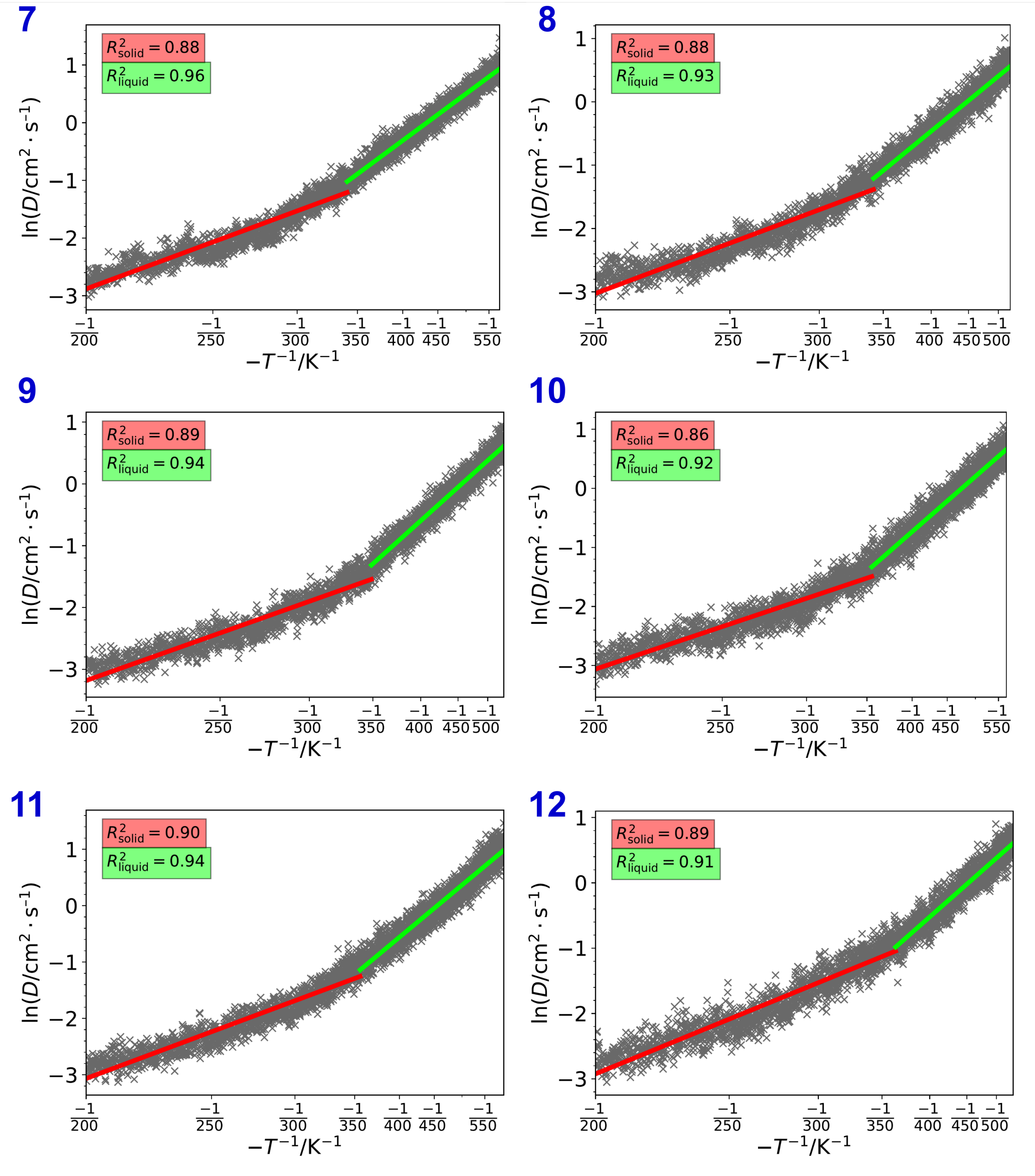

Figure S9: The diffusion coefficient (D) dependence on temperature (T) during annealing simulation for expanded ILs 7-12. The left-top number indicates the corresponding molecules in Figure S7.

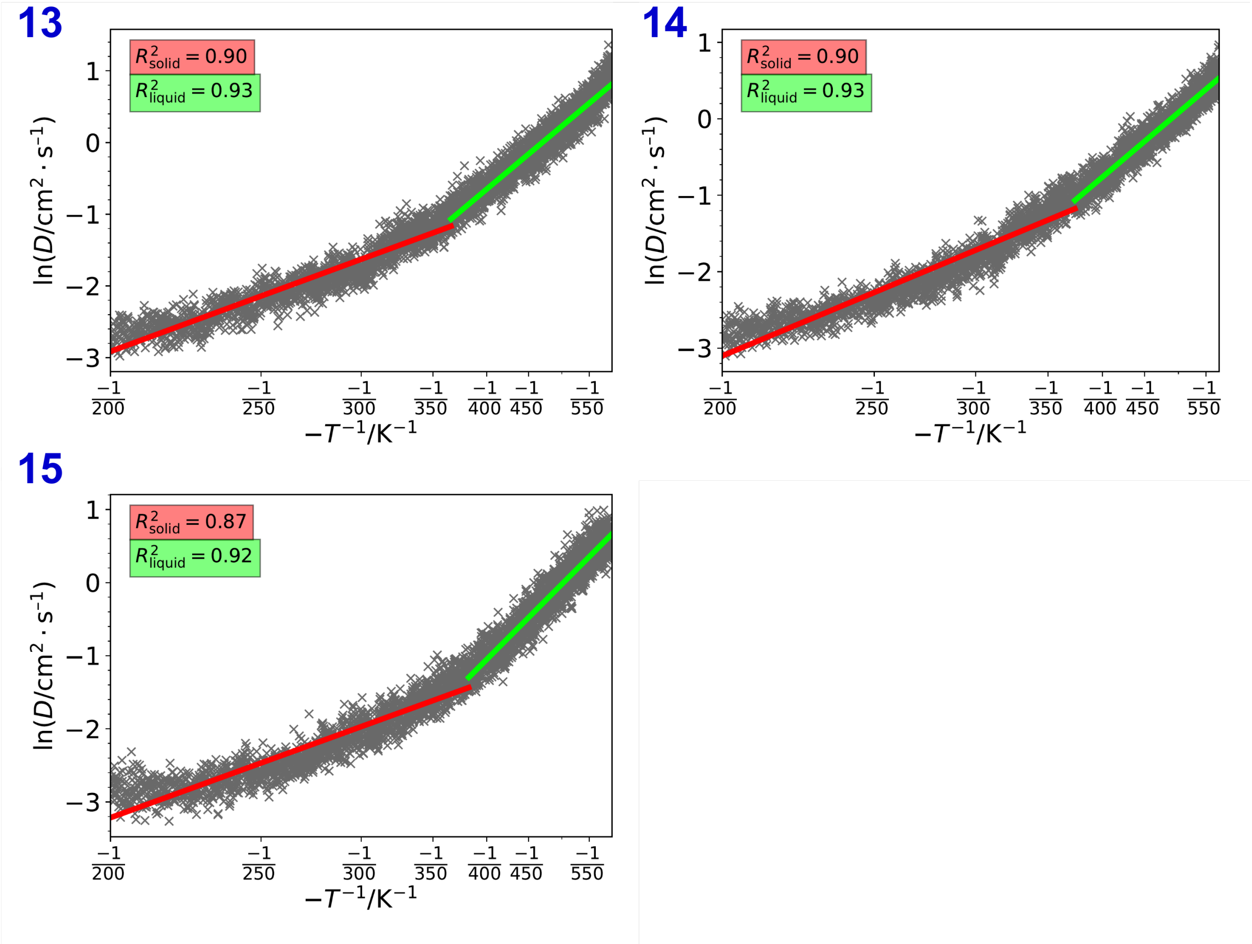

Figure S10: The diffusion coefficient (D) dependence on temperature (T) during annealing simulation for expanded ILs 13-15. The left-top number indicates the corresponding molecules in Figure S7.

References

(S1) Krzyzanowski, A.; Pahl, A.; Grigalunas, M.; Waldmann, H. Spacial ScoreA Comprehensive Topological Indicator for Small-Molecule Complexity. *J. Med. Chem.* **2023**, *66*, 12739–12750.

(S2) Ertl, P.; Rohde, B.; Selzer, P. Fast Calculation of Molecular Polar Surface Area as a Sum of Fragment-Based Contributions and Its Application to the Prediction of Drug Transport Properties. *J. Med. Chem.* **2000**, *43*, 3714–3717.

(S3) Labute, P. A widely applicable set of descriptors. *J. Mol. Graph. Model.* **2000**, *18*, 464–477.

(S4) Ren, G.; Mroz, A.; Philippi, F.; Welton, T.; Jelfs, K. Deep learning-enabled discovery of low-melting-point ionic liquids. 2025; `https://doi.org/10.26434/chemrxiv-2025-mzwd4`.

(S5) Bannwarth, C.; Ehlert, S.; Grimme, S. GFN2-xTB—An Accurate and Broadly Parametrized Self-Consistent Tight-Binding Quantum Chemical Method with Multipole Electrostatics and Density-Dependent Dispersion Contributions. *J. Chem. Theory Comput.* **2019**, *15*, 1652–1671.

(S6) Martínez, L.; Andrade, R.; Birgin, E. G.; Martínez, J. M. PACKMOL: A package for building initial configurations for molecular dynamics simulations. *J. Comput. Chem.* **2009**, *30*, 2157–2164.
